# Everything Matters: The ReproNim Perspective on Reproducible Neuroimaging

**DOI:** 10.3389/fninf.2019.00001

**Published:** 2019-02-07

**Authors:** David N. Kennedy, Sanu A. Abraham, Julianna F. Bates, Albert Crowley, Satrajit Ghosh, Tom Gillespie, Mathias Goncalves, Jeffrey S. Grethe, Yaroslav O. Halchenko, Michael Hanke, Christian Haselgrove, Steven M. Hodge, Dorota Jarecka, Jakub Kaczmarzyk, David B. Keator, Kyle Meyer, Maryann E. Martone, Smruti Padhy, Jean-Baptiste Poline, Nina Preuss, Troy Sincomb, Matt Travers

**Affiliations:** ^1^Eunice Kennedy Shriver Center, Department of Psychiatry, University of Massachusetts Medical School, Worcester, MA, United States; ^2^McGovern Institute for Brain Research, Massachusetts Institute of Technology, Cambridge, MA, United States; ^3^TCG, Inc., Washington, DC, United States; ^4^Department of Neuroscience, University of California, San Diego, San Diego, CA, United States; ^5^Department of Psychological and Brain Sciences, Dartmouth College, Dartmouth, NH, United States; ^6^Institute of Psychology, University of Magdeburg, Magdeburg, Germany; ^7^Department of Psychiatry and Human Behavior, University of California, Irvine, Irvine, CA, United States; ^8^Department of Neurology and Neurosurgery, McGill University, Montreal, QC, Canada

**Keywords:** reproducibility, neuroimaging, data model, publication, re-executability

## Abstract

There has been a recent major upsurge in the concerns about reproducibility in many areas of science. Within the neuroimaging domain, one approach is to promote reproducibility is to target the re-executability of the publication. The information supporting such re-executability can enable the detailed examination of how an initial finding generalizes across changes in the processing approach, and sampled population, in a controlled scientific fashion. ReproNim: A Center for Reproducible Neuroimaging Computation is a recently funded initiative that seeks to facilitate the “last mile” implementations of core re-executability tools in order to reduce the accessibility barrier and increase adoption of standards and best practices at the neuroimaging research laboratory level. In this report, we summarize the overall approach and tools we have developed in this domain.

## Introduction

There has been a recent major upsurge in the concerns about reproducibility in many areas of science (Ioannidis, 2005,Ioannidis, 2011; Button et al., 2013). The reasons for the concern are numerous, and there are numerous practices in the scientific field that have been found to exacerbate the problem. At a high level, a premium is put on novel, high-profile publications (in contrast to replications and negative findings) and a specific *p-*value (typically 0.05) as a proxy for truth has been adopted (Simonsohn et al., 2014; Wasserstein and Lazar, 2016). These aspects, in the context of a scientific reporting system that is out of touch with the digital age, have combined to create a perfect storm of practices that do not readily support the transparency needed to embrace reproducibility more substantively (Martone, 2015; Starr et al., 2015).

**FIGURE 1:**
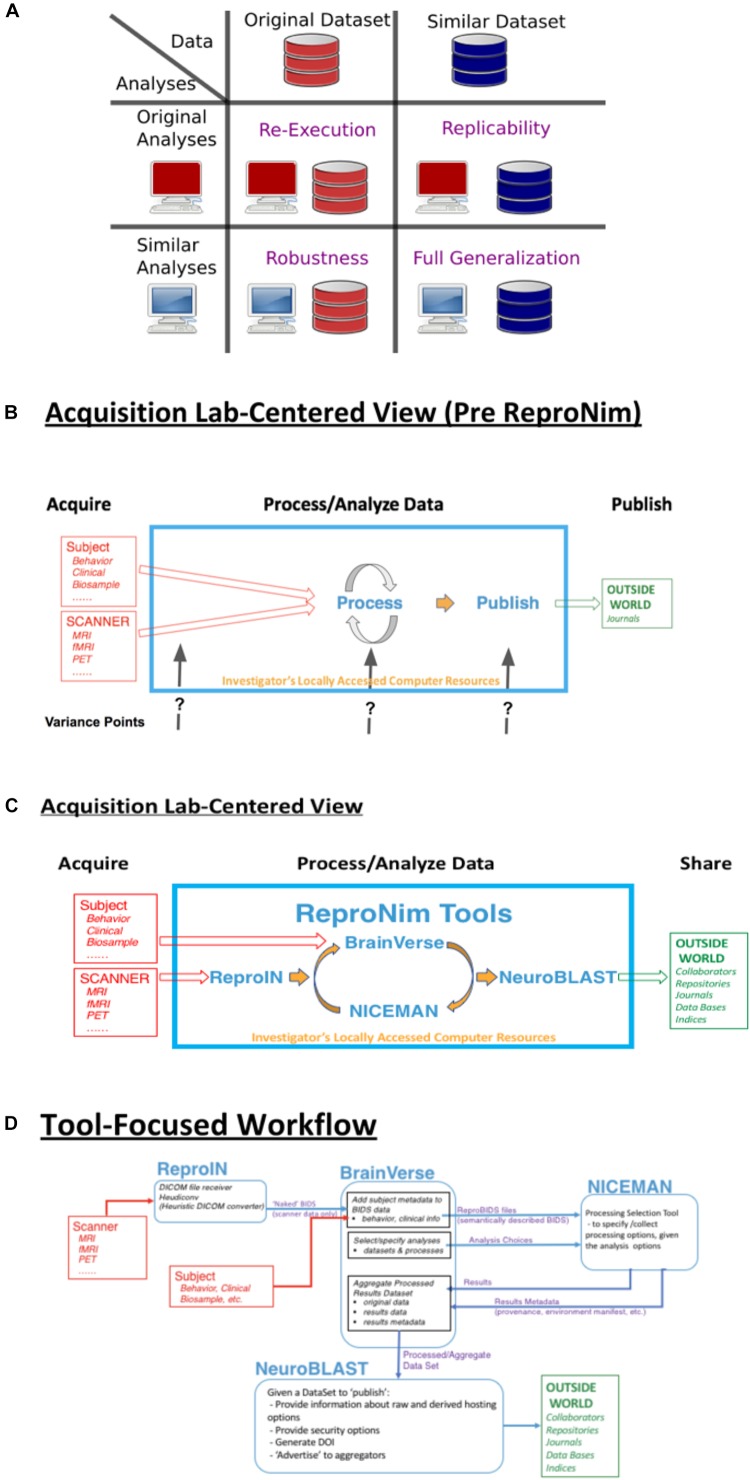
ReproNim conceptual workflows. **(A)** Pictorial depiction of the concepts of re-executability (same data, same analysis), replication (same analysis, similar data), robustness (same data, similar analysis), and generalization (similar data, similar analysis). Adapted from multiple sources, including Dr Drummond (2009), Peng (2011); Hong (2015), Goodman et al. (2016); Whitaker (2016), and Allard (2018). **(B)** General neuroimaging data workflow: Imaging data and behavioral/clinical measures enter into a local analysis, generate results that then get published. Substantial variability in the published literature exists in how the data, analysis and results are described. **(C)** The ReproNim vision of the general neuroimaging data workflow where control of the data model and machine-readable markup is invoked to completely represent the data workflow, processing and results using the tools of ReproIn, BrainVerse, NICEMAN, and NeuroBlast. **(D)** Detailed data transformations and markup as the data work their way through the planned analysis and tools.

In acknowledgment of this situation, each scientific field is forced to re-examine the best-practices that are expected of practitioners in that field. Each field grapples with what reproducibility looks like within the context of that field. Neuroimaging provides a lens on various biological processes, and how these biological processes change over the course of development, and in the face of pathological insult. As the biological process is the ultimate target of the neuroimaging inquiry, the question of reproducibility relates principally to the conclusions reached about such processes. A true biological inference about a population or process should generalize to other valid ways of observing that process and other samples of that population. In the quest to advance the overall reproducibility of neuroimaging science, one approach is to target the re-executability of the publication; the basic, current building block of the dissemination of scientific knowledge. The information supporting such re-executability can enable the detailed examination of how an initial finding generalizes across changes in the processing approach, and sampled population, in a controlled scientific fashion (see Figure 1A). It is only in the context of a systematic ability to probe a finding that the true generalizability of a claim can emerge.

It can be argued that “everything matters” in the generalizability of the traditional neuroimaging publication. The issues already identified span all levels of the experimental ecosystem:

• Computational environments matter (Glatard et al., 2015);
• Tool selection matters (Tustison et al., 2014; Dickie et al., 2017);
• Tool version matters (Dickie et al., 2017);
• Statistical model matters (Tan et al., 2016);
• Study population characteristics matter.

In the context of all these things that matter, what is an appropriate approach that investigators in this field should take? Our position is that the key to a comprehensive understanding of the published neuroimaging literature is to comprehensively, and in a machine-accessible manner, describe each of the elements of the experiment: input data, processing steps, computational environment, statistical assessment, and complete results (Ghosh et al., 2017a). The human understandable interpretations and claims, typical of a publication, can then exist around these machine-readable [and hence Findable, Accessible, Interoperable and Reusable (i.e., FAIR; Wilkinson et al., 2016)] elements. The existence of this machine readable and actionable *provenance* (the description of the origins of all elements of the publication) is what is needed to trace back and validate the underpinnings of a claim, and the starting point for the systematic examination of that claims’ generalizability.

Within the neuroimaging community, the prognosis for the ability to establish a complete description of accessible elements for all parts of the publication is quite good. The field has good data standards [DICOM^1^, NIfTI^2^, BIDS^3^ (Gorgolewski et al., 2016), MINC^4^, etc.], excellent platforms for the sharing of code and data management and sharing (Git, GitHub, DataLad, OSF, etc.), there are ample raw data repositories (XNAT^5^ (Herrick et al., 2016), NITRC-IR^6^ (Kennedy et al., 2016), NIMH Data Archive (NDA)^7^, International Neuroimaging Data-sharing Initiative (INDI)^8^ (Mennes et al., 2013), Human Connectome Project (HCP)^9^ (Marcus et al., 2013), OpenNeuro^10^, etc.), numerous workflow systems (Nipype^11^ (Gorgolewski et al., 2011), LONI Pipeline^12^ (Rex et al., 2003), etc.), package and execution management systems (NeuroDebian^13^, Docker^14^, NeuroDocker^15^, Singularity^16^, NITRC-CE^17^, etc.), and several outlets to disseminate results (NeuroVault^18^, BrainSpell^19^, NeuroSynth^20^, etc.). Importantly, a standard data model for the description of all these research elements, the Neuroimaging Data Model (NIDM)^21^ (Keator et al., 2013), is also in place to facilitate and distribute semantically annotated and unambiguous representations of the complete experimental cycle. As such, the main barrier to the generation of re-executable publications which foster reproducibility and generalizability is not the core resources, but rather the ease of use alongside the acceptance of best practices (Eglen et al., 2017; Nichols et al., 2017), in the typical neuroimaging laboratory. In addition to knowing that the resources for reproducibility exist, the community needs to embrace an approach of “Reproducible by Design” (as opposed to reproducibility as an afterthought). ReproNim: A Center for Reproducible Neuroimaging Computation is a recently funded initiative that seeks to facilitate the “last mile” implementations of these core tools in order to reduce the accessibility barrier and increase adoption of standards and best practices at the research laboratory level.

## Repronim Approach

In the remainder of this report, we provide an annotated perspective on the ReproNim vision for the re-executable publication. For this purpose, we concentrate on a laboratory data acquisition centric version of the research workflow. Other workflows (i.e., data query from accessible data resources) can be envisioned, but will be outside the purview of this report. Figure 1B depicts a stylized version of the data workflow in a typical neuroimaging experiment. Current publication practice focuses on human readable descriptions of the detailed data collection, the processing workflow and environment, and the statistical procedures and results. Therefore, across the field, there is vast variability in the detail, precision and completeness of these published descriptions. This variance in description may contribute to the limited ability of the field to replicate findings. Because we do not know exactly what a given paper did or observed, when a subsequent paper examines a similar topic it is impossible to parse similarities and differences in results appropriately. Figure 1C overviews the ReproNim vision for taking control of these variance points, through instrumentation that generates machine-readable provenance in each of the following areas: experimental data description and versioning (NIDM-E), processing workflow (NIDM-WF), and results (NIDM-R). While the analytic processing steps for a neuroimaging workflow using any processing tool (SPM^22^, FSL^23^, FreeSurfer^24^, AFNI^25^, etc.) will remain identical and completely under the researcher’s control, we will insert simple “wrapper” functionality that manage the conversion and markup of incoming imaging data (ReproIn), markup of subject-specific observations and experiment-specific analysis plans (BrainVerse), interrogation and management of execution environments (NICEMAN), and the distribution of the results to user-identified, appropriate and FAIR data repositories (NeuroBLAST). The data transformations and annotations that these tools impart upon the data flow are illustrated in Figure 1D.

## Materials and Methods

In this section we will review the current status of the key tools that are in place to support the re-executable publication. Each resource will be summarized in terms of its purpose, how to access it, and its functionality as of this writing.

### ReproIn

ReproIn is a specification and a software platform to fully automate acquisition, preparation and layout of collected MRI data in the BIDS data structure with DataLad version management, so they will be ready for local distribution and processing in a scalable and flexible manner, while retaining all provenance information from the moment of their creation, in order to ease later sharing or publication.

ReproIn is accessed from the ReproIn Github repository^26^.

To not reinvent the wheel, the software development of ReproIn is largely done through contribution to existing software projects: *HeuDiConv*^27^ – a flexible DICOM converter for organizing brain imaging data into structured directory layouts; and *DataLad*^28^ – a modular version control platform and distribution for both code and data including entire containerized computation environments via the DataLad-containers extension and automated execution provenance recording within version control systems (VCS) using DataLad’s “run” functionality to provide a fully re-executable VCS-tracked analysis record. The ReproNim project actively contributes to those existing solutions to provide all necessary components for computationally reproducible research.

General features of ReproIn include:

• A flexible naming convention for study description and acquisition details to be used at an MR scanner console that extends the information typically available in DICOM metadata, to allow for an automated translation of MR scans in DICOM format into BIDS datasets.
• A HeuDiConv ReproIn heuristic implementation^29^ to process and validate the above BIDS specification^30^.
• Support for automated metadata generation by HeuDiConv (e.g., to tag potentially sensitive information) using DataLad’s metadata capabilities.
• Datasets can be incrementally expanded with new acquisitions, as well as merged with any changes (new data, adjusted templates) from the data acquisition server.
• Optional automatic obfuscation of time stamps in the VCS records to protect privacy of study participants.
• Standalone Docker and Singularity containers for turnkey processing and analysis deployment.

### BrainVerse

BrainVerse is a cross-platform software framework and collaborative desktop application to help researchers annotate the research workflow from experimental planning to execution of analysis. Annotation includes semantic coding of all data elements, as well as the merging of the imaging data and behavioral/clinical data streams, resulting in semantically marked up BIDS data structures (the so called “ReproBIDS” datasets) also under DataLad version management. Key application areas include:

• Harmonization with the NIMH Data Archive (NDA): Allows importing and curating the NDA schemas to generate collection instruments that support harmonization of variables within and across project.
• Project planner and executor: Allows creating a plan for an experimental protocol in a project and collecting data using harmonized and reusable forms.
• NIDM term editor: Allows the community to search for and build a common descriptive vocabulary around neuroimaging.

BrainVerse is accessed from the BrainVerse website/Github repository^31^.

General features include:

• GitHub based login and authorization;
• Cross-platform support (based on the Electron^32^ framework) on desktops with an option for server based installation;
• NDA harmonizer and editor:
  ° Import, select, and preview forms,
  ° Edit forms,
  ° Push to common repository on ReproNim,
  ° Pull curated forms from ReproNim repository;
• Project planner and form-based data collector:
  ° Create project execution plan with multiple session support,
  ° Reuse/Create session instruments,
  ° Add participants to project and collect data using project plan,
  ° Export collected data to CSV files for visualization and analysis;
• NIDM term editor:
  ° Display of terms from NIDM owl files,
  ° Edit terms and send review requests.

### NICEMAN

NICEMAN is a specification and software system that supports the management of computation environments and computations, targeting the neuroimaging domain. It provides:

• A specification to describe environments consistently across available data and software distributions (e.g., VCS such as git, Debian-based systems, Conda Python distribution, Singularity and Docker images),
• A software platform to allow convenient discovery, description, and management of the computation environment(s) so that they could be easily traced (to automatically collect the specification from existing environment or a dedicated process), validated (to satisfy necessary requirements), compared (to determine how requirements differ), satisfied (to create a new or adjust an existing environment), execute necessary computation(s) and interface the output(s).

NICEMAN developed openly and accessible on Github^33^.

General features include:

• *retrace* command allows users to establish a detailed description of the environment given an initial specification (e.g., from reprozip^34^, Nipype’s.trig PROV) or from a list of files provided on the command line. It generates tracing information that is sufficient for re-establishing the environment (origins, versions, etc.) for Debian-based systems, VCS (svn and git), and Conda,
• Support of Docker, shell (via ssh or localhost) environments for scripted or interactive sessions, with a centralized resources manager (ls command to list available environments/resources and query their status), and with basic support for Docker and Amazon Web Services (AWS) backends life-time (bootup/shutdown),
• Create/install commands to fulfill the specification and provide the requested environment (via Docker, AWS, etc.).
• Support for Singularity and Docker environments.

### NeuroBlast

NeuroBlast is a share, search and discovery service. The NeuroBlast service facilitates data sharing (raw and results) of known existing repositories and assists users in the data discovery process to find matching/similar studies based on a combination of task, analysis, and activation patterns. This novel environment utilizes all information about a study, enabling researchers to select appropriate sharing sites, and find similar studies utilizing a number of different similarity metrics. This service employs deep semantics, building from terminologies managed by InterLex and its associated ontology, to enhance the search for similar data sets utilizing multiple features for comparison.

InterLex can be accessed at InterLex.org and the ontology can be accessed from its GitHub repository^35^.

## Results

In this section, we briefly summarize a couple of example use-cases that demonstrate the ReproNim vision in action.

### Tools Matter

Shared neuroimaging data is an important means of promoting an open and reproducible neuroimaging analysis culture. The Autism Brain Imaging Data Exchange (ABIDE1) dataset (Di Martino et al., 2014) is a premier example of shared neuroimaging data that promotes exploration of the factors related to the autism diagnosis relative to features accessible in structural and resting state functional MRI in over 1000 subjects. There are many factors related to the reproducibility of neuroimaging findings, including selection of software tools. In this report, we take advantage of the ABIDE Preprocessed Connectomes project^36^ which has performed a comparative analysis of ABIDE1 data using three widely used structural analysis software tools: FreeSurfer (Fischl et al., 2002), versions 5.1 and 5.3, and ANTS (Avants et al., 2011). In an ideal world, regional thickness data would be independent of the specific software tool used to generate the result, when applied to common data. We utilize this dataset to evaluate the extent to which the selection of a software tool matters, and provide a common open source platform to support further exploration of these results. We identified the subset of (976 cases (from the 1112 ABIDE1 original cases)) that had completed all three analyses and are available at the ABIDE Preprocessed Connectomes site.

**FIGURE 2:**
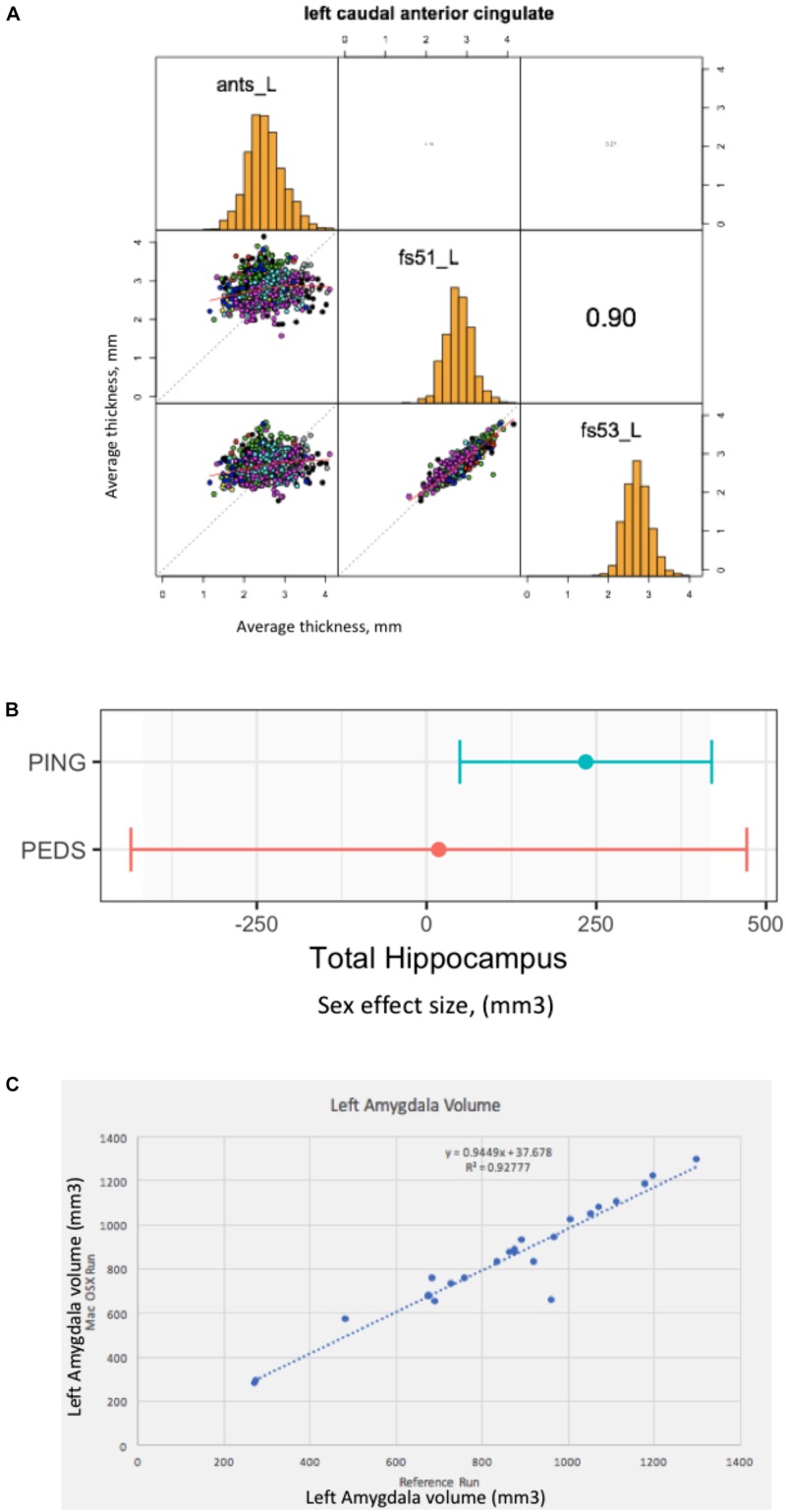
Everything matters. **(A)** Tools matter: Same data (976 ABIDE1 cases), different tools (FreeSurfer 5.1 and 5.3 and ANTS). For a specific anatomic region (left caudal anterior cingulate cortex), we show a matrix of the between tool comparisons. On the diagonal (from upper left to lower right) we see the distribution histogram of average left caudal anterior cingulate cortex thicknesses for ANTS, FreeSurfer 5.1 and FreeSurfer 5.3, respectively. The three scatter plots (left column, middle, left column bottom and middle column bottom) show the between tool scatter plots and regression line for these data for: ANTS vs. FreeSurfer 5.1 (Pearson’s correlation coefficient *r* = 0.16); ANTS vs. FreeSurfer 5.3 (Pearson’s correlation coefficient *r* = 0.21); and FreeSurfer 5.1 vs. FreeSurfer 5.3 (Pearson’s correlation coefficient *r* = 0.90), respectively. **(B)** Sample size matters: Same analysis (FreeSurfer 5.3 and a statistical model looking at gender effects in hippocampus volume) as a function of the large-scale publically available structural imaging data in typically developing children in ∼2005 (NIH PEDS, *N* = 325) and ∼2011 (PING, *N* = 1239). The plot shows the observed effect size and 95% confidence interval for the total hippocampal volume for these two cohorts. **(C)** Computational Environment Matters: Same data, same workflow, different workflow operating system environments results in different results, as shown for the volume of the left amygdala in subset of 24 cases. See text for further details.

The result of this effort is a publically available GitHub repository^37^, which identifies the specific cases that are included, contains summary data tables of the volume and surface area results of the three analysis tools, software to load these data tables into the R statistical software analysis package (R reader), and an R script to correlate the corresponding analytical results between the different structural analysis runs. The surface-based results are represented as average cortical thickness for each of the 62 (31 bi-laterally represented) anatomic regions in the Desikan-Killiany-Tourville (DKT) atlas (Klein and Tourville, 2012). For each anatomic region, we calculate the three inter-tool result correlations (FreeSurfer 5.1 vs. FreeSurfer 5.3; FreeSurfer 5.1 vs. ANTS, and Freesurfer 5.3 vs. ANTS). Findings can be summarized as follows. The mean and range of region-wise correlation were observed as follows between the various tool-pair combinations: ANTS vs. FreeSurfer 5.1 mean regional correlation = 0.43, [minimum = 0.19 (rostralanteriorcingulate L), maximum = 0.59 (superiortemporal R)]; ANTS v FreeSurfer 5.3 mean regional correlation = 0.47, [minimum = 0.19 (caudalanteriorcingulate R), maximum = 0.67 (superiortemporal R)]; FreeSurfer 5.1 vs. FreeSurfer 5.3 mean regional correlation = 0.87, [minimum = 0.76 (insula R), maximum = 0.93 (paracentral L)]. The FreeSurfer analysis in this data presents excellent inter-version (5.1–5.3) commonality. There are, however, substantial differences between the regional thickness results between the FreeSurfer and ANTS analysis. As an example, the scatter plots and distributions for the left caudal anterior cingulate is shown in Figure 2A.

### Sample Size/Quality Matters

In this example, we look at the potential gender effect of total hippocampal volume in typically developing children, and how an observation of this effect can evolve over time as a function of the imaging technology and the amount of available data. We model total hippocampus volume as a function of gender, covarying for age, sex by age interaction, site, and total cerebral volume. We used state-of-the-art at the time data available from two national typically developing cohorts. We first look at the gender effect as observed in ∼2005 from the NIH Pediatric Database (*N* = 325 (159 males/166 females); aged 4.2–18.4 years) (Evans and Brain Development Cooperative Group, 2006). We also look at data from the PING cohort (Pediatric Imaging, Neurocognition and Genetics) (Jernigan et al., 2016), as released in ∼2011 (total *N* = 1239 (644 males/595 females), aged 3–20 years). We applied a common analysis (FreeSurfer 5.3) using default parameters to each of these datasets in house. These results are shown in Figure 2B. In this case, we note a lack of significant gender dimorphism of the total hippocampus seen in children from the PEDS cohort (*p* = 0.9379). However, the PING dataset documents a significant gender effect for the total hippocampus volume (*p* = 0.013269). While sample size is one of the differences between these studies, it is also the case the image quality and acquisition technology had evolved in the years between these two studies. Nevertheless, we feel that this type of observation is reflects the types of conclusions that are often gleaned from the literature: observations that are not significant based upon older, smaller N studies may not generalize to newer, larger *N* studies. The tightening of the error bars around a specific observation can be attributed to many sources, not the least of which, in this case is the sample size. Indeed, the observed effect size in the PING sample falls within the observed range of the older, smaller PEDS distribution of observations.

### Simple Re-executable Publication

In this last example, we document a set of procedures, which include supplemental additions to a manuscript, that unambiguously define the data, workflow, execution environment and results of a neuroimaging analysis, in order to generate a verifiably re-executable publication. Re-executability provides a starting point for examination of the generalizability and reproducibility of a given finding. We have provided an example “publication” with four supplementary files (Ghosh et al., 2017a), the: (1) data file, (2) workflow file, (3) execution environment specification, and (4) results. In this example, the data is from 24 publically accessible typically developing subjects between the ages of 10–15 that have a structural scan at 3 Tesla available from the 1000 Functional Connectomes Project at NITRC (doi 10.18116/C6C592; Kennedy, 2017). The workflow is a FSL-based (version 5.0.9) assessment of total brain, gray and white matter and subcortical structural volumes and is accessible at doi: 10.5281/zenodo.800758, (Ghosh et al., 2017b). The execution environment is controlled through the use of Docker; the docker image is available at https://github.com/ReproNim/simple_workflow. Finally, the complete results of the reference run are stored in the expected_output folder of the GitHub repository^38^. By sharing the results of this reference run, as well as the data workflow, and a program to compare results from different runs, we can enable others to verify that they can arrive at the exact same result (if they use the exact same workflow and execution environment), or how close they come to the reference results if they utilize a different computational system (that may differ in terms of operating system, software versions, etc.). Figure 2C demonstrates the imprecision of “the same data and workflow” run (in this case left amygdala volumes for each of the 24 subjects) on different hardware platforms (Docker Debian 8.7 (Reference Run) vs. Mac OS X 10.12.4), documenting the importance of taking control over the complete description of all elements of the reported research publication. Ideally, while the amygdala volume will differ by subject, the same workflow when rerun should yield the line of identity. It is the case that when the same Docker image is run, the identical results are generated. However, as illustrated in Figure 2C, running the same workflow on a Debian 8.7 vs. Mac OS X 10.12.4 system the results deviate substantially from the expected relationship.

## Summary

In this perspective we have reviewed the ReproNim vision and rationale for enhancing the reproducibility of the neuroimaging literature through an emphasis on individual publication re-executability. A given publication, if published in a completely re-executable fashion, forms the basis for future systematic explorations of the generalization of the observations through independent manipulation of the data and processing details separately. Reproducible claims and conclusions are supported by findings that are generalizable to data beyond that originally reported and should be demonstrated to be robust with respect to details of the analytic approach. The key to controlling the re-executability of the publication is the generation and reporting, at all stages of the process, machine readable provenance documentation that details the input data sources, the analysis workflow, the statistical model, the execution environment and the complete results. Since we know that all these factors matter, a good scientific report should be able to describe each of these factors unambiguously.

Time will tell if the tools and procedures promoted by the ReproNim effort (or other efforts) to enhance publication level re-executability will be successful. We can assert that the majority of neuroimaging publications to date do not expose this complete set of publication details explicitly. We envision a future re-executability check list that can be retrospectively applied by the community to the corpus of publications (or, better yet, used by reviewers of publications prospectively) that generates a catalog of compliant elements on a publication by publication basis. One can then observe, over time, the extent to which the exposure of publication elements (input data, workflow, execution environment, complete results) increases. Efforts are underway to generate more compelling scientific examples of the re-executable publication in response to exploring the generalizability of specific findings in the autism and schizophrenia literature.

## Author Contributions

All authors participated in the conception of the project, the development of various software aspects and writing of this manuscript.

## Conflict of Interest Statement

AC, NP, and MT are employed by TCG, Inc. The remaining authors declare that the research was conducted in the absence of any commercial or financial relationships that could be construed as a potential conflict of interest.

## Footnotes

1 https://www.dicomstandard.org/current/

2 https://nifti.nimh.nih.gov/nifti-1/

3 http://bids.neuroimaging.io/

4 https://en.wikibooks.org/wiki/MINC/SoftwareDevelopment/MINC2.0_File_Format_Reference

5 https://www.xnat.org/

6 https://www.nitrc.org/ir/

7 https://data-archive.nimh.nih.gov/

8 http://fcon_1000.projects.nitrc.org/

9 https://www.humanconnectome.org/

10 https://openneuro.org/

11 https://nipype.readthedocs.io/en/latest/

12 http://pipeline.loni.usc.edu/

13 http://neuro.debian.net/

14 https://www.docker.com/

15 https://github.com/kaczmarj/neurodocker

16 https://www.sylabs.io/docs/

17 https://www.nitrc.org/ce/

18 https://neurovault.org/

19 http://brainspell.org/

20 http://neurosynth.org/

21 http://nidm.nidash.org/

22 https://www.fil.ion.ucl.ac.uk/spm/

23 https://fsl.fmrib.ox.ac.uk/fsl/fslwiki

24 https://surfer.nmr.mgh.harvard.edu/

25 https://afni.nimh.nih.gov/

26 https://github.com/ReproNim/reproin

27 https://github.com/nipy/heudiconv

28 https://www.datalad.org/

29 https://github.com/nipy/heudiconv/blob/master/heudiconv/heuristics/reproin.py

30 https://github.com/nipy/heudiconv/blob/master/heudiconv/heuristics/reproin_validator.cfg

31 https://github.com/ReproNim/brainverse

32 https://electronjs.org/

33 https://github.com/repronim/niceman

34 https://www.reprozip.org/\#

35 https://github.com/SciCrunch/NIF-Ontology

36 http://preprocessed-connectomes-project.org/abide/

37 https://github.com/companat/compare-surf-tools

38 https://github.com/ReproNim/simple_workflow/tree/1.1.0/expected_output
